# Incessant Automatic Atrial Tachycardia in a Neonate Successfully Treated with Nadolol and Closely Spaced Doses of Flecainide: A Case Report

**DOI:** 10.3390/pediatric12030024

**Published:** 2020-11-11

**Authors:** Gilda Belli, Mattia Giovannini, Giulio Porcedda, Marco Moroni, Giancarlo la Marca, Guglielmo Capponi, Silvia Favilli, Luciano De Simone

**Affiliations:** 1 Department of Health Sciences, Post-graduate School of Pediatrics, Meyer Children’s University Hospital, University of Florence, Viale Pieraccini 24, 50139 Florence, Italy; mattia.giovannini@unifi.it (M.G.); guglielmo.capponi@unifi.it (G.C.); 2 Department of Pediatric Cardiology, Meyer Children’s University Hospital, Viale Pieraccini 24, 50139 Florence, Italy; giulio.porcedda@meyer.it (G.P.); silvia.favilli@meyer.it (S.F.); luciano.desimone@meyer.it (L.D.S.); 3 Neonatal Intensive Care Unit-Medical Surgical Fetal-Neonatal Department, Meyer Children’s University Hospital, Viale Pieraccini 24, 50139 Florence, Italy; marco.moroni@meyer.it; 4 Department of Experimental and Clinical Biomedical Sciences, Meyer Children’s University Hospital, University of Florence, Viale Pieraccini 24, 50139 Florence, Italy; giancarlo.lamarca@unifi.it

**Keywords:** supraventricular tachyarrhythmia, automatic atrial tachycardia, flecainide, pharmacokinetics, newborn

## Abstract

Supraventricular tachyarrhythmia (SVT) is the most common type of arrhythmia in childhood. Management can be challenging with an associated risk of mortality. A female neonate was diagnosed with episodes of SVT, controlled antenatally with digoxin. Flecainide was commenced prophylactically at birth. Despite treatment, the infant developed a narrow complex tachycardia at 5 days of age. The electrocardiogram features were suggestive of either re-entry tachycardia or of automatic atrial tachycardia (AAT). Following several unsuccessful treatments, a wide complex tachycardia developed. A transesophageal electrophysiological study led to a diagnosis of AAT. Stable sinus rhythm was finally achieved through increasing daily administrations of flecainide up to six times a day, in association with nadolol. The shortening of intervals to this extent has never been reported before and supports the evidence of a personal, age-specific variability in pharmacokinetics of flecainide. Larger studies are needed to better define the appropriate dose and timing of administration.

## 1. Introduction

Supraventricular tachyarrhythmias (SVT) is the commonest type of arrhythmia in the pediatric population [1]. Among these, the most frequent is by far atrioventricular re-entry tachycardia (AVRT), caused by the presence of an accessory pathway between the atria and ventricles creating a re-entry circuit. In contrast, automatic atrial tachycardia (AAT), a rare form of SVT (6–10% of SVT in infants <1 year old) [1] is caused by an increased automaticity of a non-sinus atrial site and is often resistant to pharmacologic treatment [2,3] occurring in incessant form [3,4]. Younger patients with arrhythmia have a higher risk of related heart failure (tachycardiomyopathy) and it is therefore imperative to achieve early stabilization of the heart rate [2,4]. Diagnosis and management can be challenging and the mortality rate in AAT is around 2% [5].

The following case is the first reported case of a newborn with incessant atrial tachycardia, which was resistant to several anti-arrhythmic drugs, in which a stable control of the heart rhythm was finally achieved with the combination of nadolol and frequent administrations of flecainide [2].

## 2. Case Report

The clinical history began at 30 weeks of gestational age, when an obstetric echography discovered a fetal SVT at 250 beats per minute (bpm). Initially the mother was administered digoxin (for about 5 days) and subsequently flecainide which achieved a stable cardioversion lasting until the end of pregnancy. 

The neonate was born at 35 weeks + 2 days of gestational age by induced vaginal delivery due to pre-eclampsia, with a body weight at birth of 2450 g. An electrocardiogram (ECG) confirmed sinus rhythm and an echocardiogram showed good biventricular function and absence of congenital heart disease. There was no family history of any structural and arrhythmic cardiac diseases. However, given the clinical history, flecainide (50 mg/m^2^/day, twice a day) was administered prophylactically at birth.

**Figure 1:**
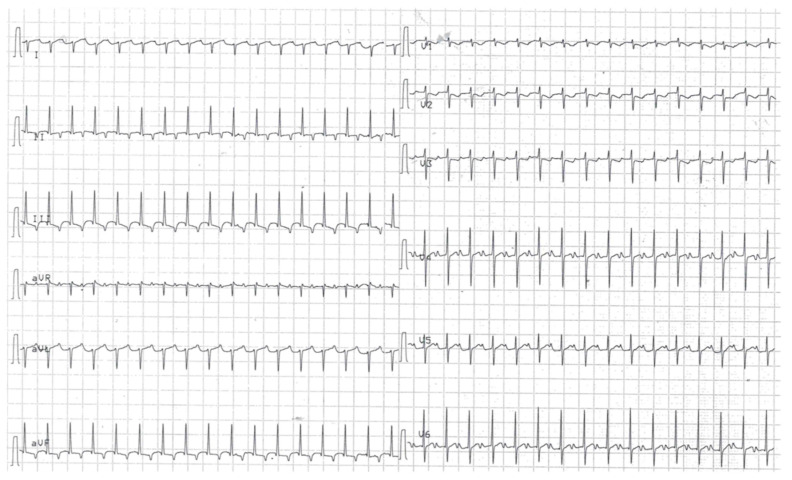
Surface electrocardiogram showing narrow QRS tachycardia with negative P waves inscribed at the end of T waves in D2, D3, aVF. RP’ (160 ms) is longer than P’R (140 ms), PR is constant.

At 5 days of life, after a crying episode, a narrow QRS complex tachycardia was detected at 210 bpm. A 12-leads ECG showed a left axis deviation with negative P waves inscribed at the end of T waves in D2, D3, aVF (Figure 1) and a prolonged PR interval. Multiple episodes occurred in the following days: adenosine (0.2 mg/kg) temporarily slowed the heart rate, but a cardioversion was not achieved, even with the addition of propanolol at 2.5 mg/kg/day. Routine blood examinations were normal. Subsequently, flecainide and propanol were interrupted and intravenous amiodarone (loading dose of 5 mg/kg, followed by 10 mg/kg/day) and oral digoxin (10 mcg/kg) were started. 

**Figure 2:**
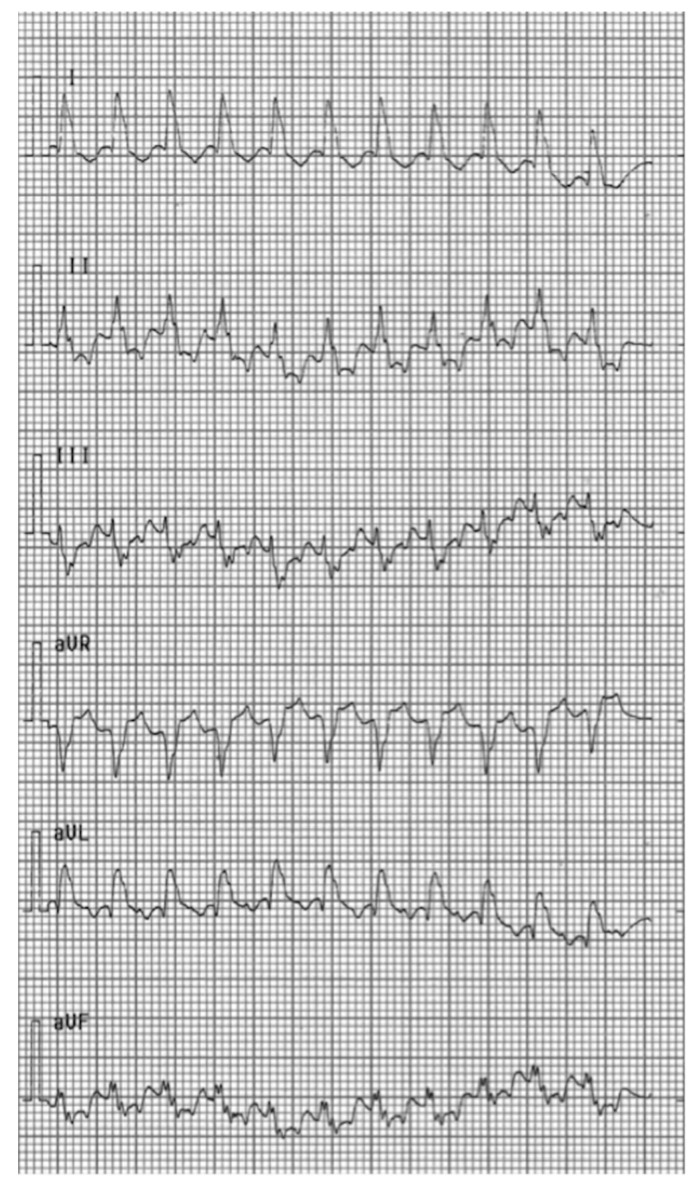
Surface electrocardiogram showing wide QRS tachycardia occurred after the addition of sotalol to treatment with flecainide.

**Figure 3:**
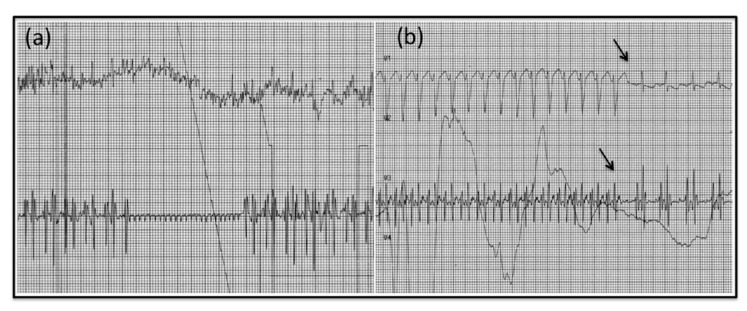
Transesophageal electrophysiological study. Lack of response to overdrive pacing (**a**) and absence of a blocked retrograde atriogram following the last beat of tachycardia (**b**) suggested automatic atrial tachycardia.

At 10 days of life, she was admitted to our hospital. Digoxin was interrupted while amiodarone was increased up to 18 mg/kg/day (i.e., 13.3 mcg/kg/min) without obtaining cardioversion, but only adequate heart rate control up to 140 bpm. Afterwards, due to persistence of tachycardia, oral flecainide was re-commenced at a higher dose of 100 mg/m^2^/day (9 mg twice a day), and intravenous amiodarone was discontinued in the following days. Blood levels of flecainide were periodically checked with blood tests. A period of prevalent sinus rhythm was achieved, however, due to relapses, the dose of flecainide was increased up to 120 mg/m^2^/day (8 mg thrice a day) and sotalol was also added at 3 mg/kg/day (3 mg thrice a day) and then increased up to 6 mg/kg/day (6 mg thrice a day). At 29 days of life, a wide complex tachycardia developed with a heart rate of 215–240 bpm, undetectable P waves (Figure 2), and with the help of a transesophageal electrophysiological study (TEES) a ventricular tachycardia (VT) was excluded and the diagnosis of automatic atrial tachycardia (AAT) was confirmed (Figure 3). 

Therefore, it was decided to switch from sotalol and flecainide to nadolol at about 4 mg/kg/day (3 mg four times a day). In the following days, the infant represented with AAT, and flecainide (80 mg/m^2^/day, 4 mg four times a day) was added to the treatment. During this combination therapy, episodes of SVT were noted to reappear just before the next administration of flecainide and they became progressively less frequent as we increased the number of daily administrations. Eventually, there were no further recurrences when flecainide was administered every 4 h, at a dosage of 130 mg/m^2^/day. Given the frequent need of adjusting doses and intervals of administrations, we routinely performed ECGs and in-hospital monitoring of plasma concentration of flecainide. Plasma levels of the drug were persistently in normal range (up to 500 ng/mL), which enabled us to obtain control of the arrhythmia without eliciting any serious adverse events.

During the admission, the infant maintained appropriate weight gain and she was discharged home at 54 days of life, with flecainide 5 mg six times a day (130 mg/m^2^/day), nadolol 5 mg twice a day and 4 mg twice a day (4.5 mg/kg/day), and ECG home monitoring.

During her first follow-up visit (at 65 days of life), an ECG showed a sinus rhythm with a heart rate of 122 bpm with good control of the arrhythmia. No further episodes of SVT were detected and no adverse effects were reported. Around four months after discharge the frequency of administrations was gradually reduced, and the dose adjusted higher as per the weight gain.

## 3. Discussion

Our patient presented with a narrow complex tachycardia, where ECG features demonstrated either a re-entry or an AAT. The presence of a prolonged PR interval, along with an RP’ interval longer than PR and negative P waves in inferior leads, suggested a permanent form of junctional reciprocating tachycardia (PJRT, Coumel type), [1] an uncommon type of AVRT using the atrioventricular node as the anterograde limb and a slowly conducting right postero-septal accessory pathway as the retrograde one. Nevertheless, an AAT could not be ruled out, since the occurrence of ectopic foci near the tricuspid annulus could be responsible for left P waves axis and for negative P waves in V1 and in the inferior leads. Moreover, PJRT is rare in neonates. Although the response of arrhythmia to the administration of an intravenous bolus of adenosine is usually useful to distinguish between re-entry and automaticity, [6] in our case it was not conclusive. However, the transesophageal atrial recording and stimulation enabled the diagnosis of AAT, since it allowed a better analysis of atriograms (Figure 3). The conserved P-QRS sequence ruled out VT and junctional ectopic tachycardia, while the evidence of a wide-QRS complex tachycardia, the refractory response to overdrive pacing, the absence of a blocked retrograde atriogram following the last beat of tachycardia (Figure 3b), overall were not consistent with PJRT. Therefore, an electrophysiological study may be useful in cases of tachycardia of uncertain origin and unsatisfactory response to medical treatment [1].

Because of AAT’s self-limiting course, pharmacological management is preferred over surgical or catheter ablation, however, there is still no large consensus about SVT’s most appropriate pharmacological treatment in infants [3,5].

In our patient, the addition of sotalol (a class III antiarrhythmic drug, which prolongs the QRS duration and is especially effective in termination of re-entry tachyarrhythmias), [7] resulted in a wide complex tachycardia, without restoration of sinus rhythm. On the contrary, we achieved a satisfactory control of AAT with the combination of flecainide and nadolol. Nadolol, being a non-selective beta-blocker, acts on diastolic depolarization of atrial cells with enhanced automaticity in ectopic foci, thus decreasing their firing rate and, consequently, reducing the heart rate. Indeed, unlike our case, beta-blockers are usually effective as monotherapy of AAT [6,8].

Flecainide is a class Ic antiarrhythmic that blocks fast sodium channels, thus slowing conduction throughout myocardium and decreasing automaticity [3]. In infants and children, it has a widespread use, since it has been proven to be safe and effective in the acute and long-term treatment of SVT [9,10,11]. Unlike in adults, dosing should be based on body surface area rather than on body weight, since the former dosing has been found to better correlate with flecainide serum trough levels [11]. The dose used in oral form for chronic therapy range from 50 mg/m^2^/day to 200 mg/m^2^/day, with an average effective dose of 140 mg/m^2^/day (corresponding to 1–23.5 mg/kg/day, average dose 4 mg/kg/day). Flecainide is usually administered every 8–12 h [2,11,12]. Except for rare cases, total daily doses should not exceed 200 mg/m^2^/day or 8 mg/kg/day, since greater doses have been shown to be proarrhythmic [3,11]. However, pharmacokinetics of flecainide in pediatric age has not been extensively evaluated. Firstly, in infants, intestinal absorption of orally administered flecainide can be reduced because of dietary products (e.g., milk, infant formula) [11]. Moreover, a large interindividual variability in systemic clearance and bioavailability of flecainide has been reported. This can be attributed to the genetic polymorphisms of CYP2D6, which metabolizes the drug in the liver, but also to functional variability in the transporters in the kidney (P-glycoprotein) and intestine (H+/tertiary amine antiporter) [13]. As far as median elimination half-life is concerned, Till et al. described a significant linear correlation between elimination half-life of flecainide and age (*r* = 0.75; *p* < 0.01), both in case of oral and intravenous treatment [14]. In the review by Perry et al., despite the evidence of age dependence in the elimination half-life of the drug, children aged 1 to 12 years had shorter elimination half-life values, about 8 h, compared with older children (mean value 11–12 h) [11]. Besides, in a previous article, the author emphasizes that further evaluation of flecainide kinetics is needed in children under 1 year of age, given the low correlation coefficient for serum trough levels and dose [12]. Moreover, little data are available for infants under the age of 1 month [11].

Interestingly, in our patient, a good control of arrhythmia and restoration of sinus rhythm was only achieved after increasing the number of daily administrations of flecainide to six times a day. The shortening of intervals to this extent has never been reported before and seems to support the reports of a lower absorption of flecainide, shorter elimination half-life and different pharmacokinetic parameters in newborns. 

## 4. Conclusions

In conclusion, the narrow therapeutic index [15], the high personalised variation in bioavailability and clearance, and the paucity of data about flecainide’s pharmacokinetics in the pediatric population, strongly highlight the importance of an individualized targeted therapy, even by means of frequent administrations. Regular monitoring of plasma flecainide concentrations is strictly required, at initiation of therapy and during any dose alterations, in order to optimize efficacy and avoid any adverse effects. Nevertheless, further larger studies are required to better define the pharmacokinetics and eventually the appropriate dosage and frequency at different ranges of age.
